# Evaluation of CT-Guided Ultra-Low-Dose Protocol for Injection Guidance in Preparation of MR-Arthrography of the Shoulder and Hip Joints in Comparison to Conventional and Low-Dose Protocols

**DOI:** 10.3390/diagnostics11101835

**Published:** 2021-10-04

**Authors:** Anja Goeller, Tobias Pogarell, Matthias Stefan May, Michael Uder, Peter Dankerl

**Affiliations:** Department of Radiology, University Hospital Erlangen, 91054 Erlangen, Germany; an-goeller@t-online.de (A.G.); matthias.may@uk-erlangen.de (M.S.M.); michael.uder@uk-erlangen.de (M.U.); peter.dankerl@uk-erlangen.de (P.D.)

**Keywords:** radiation exposure, CT intervention, radiation protection, tin-filtration, joint injection, MR-arthrography

## Abstract

To evaluate patients’ radiation exposure undergoing CT-guided joint injection in preparation of MR-arthrography. We developed a novel ultra-low-dose protocol utilizing tin-filtration, performed it in 60 patients and compared the radiation exposure (DLP) and success rate to conventional protocol (26 cases) and low-dose protocol (37 cases). We evaluated 123 patients’ radiation exposure undergoing CT-guided joint injection from 16 January–21 March. A total of 55 patients received CT-guided joint injections with various other examination protocols and were excluded from further investigation. In total, 56 patients received shoulder injection and 67 received hip injection with consecutive MR arthrography. The ultra-low-dose protocol was performed in 60 patients, the low-dose protocol in 37 patients and the conventional protocol in 26 patients. We compared the dose of the interventional scans for each protocol (DLP) and then evaluated success rate with MR-arthrography images as gold standard of intraarticular or extracapsular contrast injection. There were significant differences when comparing the DLP of the ultra-low-dose protocol (DLP 1.1 ± 0.39; *p* < 0.01) to the low dose protocol (DLP 5.3 ± 3.24; *p* < 0.01) as well as against the conventional protocol (DLP 22.9 ± 8.66; *p* < 0.01). The ultra-low-dose protocol exposed the patients to an average effective dose of 0.016 millisievert and resulted in a successful joint injection in all 60 patients. The low dose protocol as well as the conventional protocol were also successful in all patients. The presented ultra-low-dose CT-guided joint injection protocol for the preparation of MR-arthrography demonstrated to reduce patients’ radiation dose in a way that it was less than the equivalent of the natural radiation exposure in Germany over 3 days—and thereby, negligible to the patient.

## 1. Introduction

The gold standard to diagnose labral tears in hips and shoulders is MR-arthrography featuring direct contrast injection into the joint [1,2,3]. Although conventional fluoroscopy represents the most widely used imaging modality for injection guidance (61%), followed by ultrasound (26%), roughly 9% of MR-arthrographies are prepared using CT guidance for joint injection [4]. As stated by Mulligan this in many cases might be owed to a lack of a conventional fluoroscopy suite or even a sonography unit, especially in outpatient imaging centers [5] which primarily focus on cross-sectional examinations. Furthermore, although CT-guided percutaneous needle biopsy is the gold standard to probe and diagnose most types of cancer [6], it is currently being avoided whenever possible for the preparation of MR-arthrography due to increased radiation dose in contrast to conventional fluoroscopy. Almost 20 years ago Binkert et al., proposed CT fluoroscopy as an image guiding modality for shoulder injections to substantially decrease patient’s radiation dose [7]. However, this technique has the drawback of increased radiation dose to the medical staff as the interventional radiologist is exposed to ionizing radiation while injecting the needle into the shoulder joint during CT fluoroscopy. Over the course of a work life this has a share in the accumulation of the occupational dose radiologists receive—with still unknown long term effects on health [8]. Therefore, similar to the workflow of most other CT-guided interventions, [9,10] CT image guidance without fluoroscopy remains the predominant workflow for joint injections in clinical practice. In order to decrease patients’ radiation dose when undergoing CT-arthrography, various approaches have been taken recently, such as decreasing the tube potential of the X-ray tube [11], utilization of tin-filtration [12] or C-arm flat-panel CT-arthrography [13]. However, to our knowledge until now nobody investigated how to substantially decrease radiation dose for patients and medical staff when performing CT-guided joint injection as preparation for MR-arthrography. Roughly 29% of MR-arthrographies are indicated to suspected labrum defects (in hip and shoulder) and 6% [4] for evaluation of femoroacetabular impingement. This particular patient population usually is in the range of 20–40 years, making the reduction of the radiation dose for the preparation of MR-arthrography highly beneficial to the patient. Therefore, we developed a CT-guided joint injection protocol utilizing tin filtration and aggressive X-ray spectral shaping to drastically reduce radiation dose.

**Figure 1:**
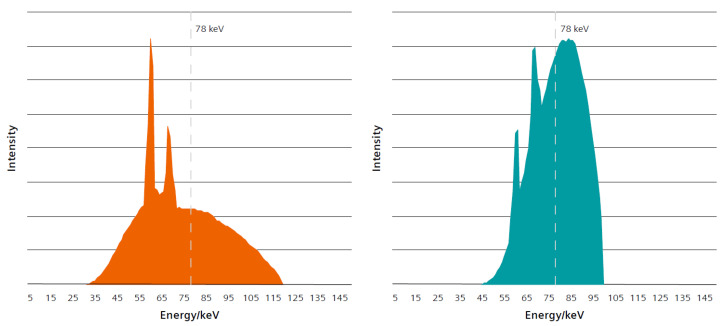
The 120 kV spectrum with standard pre-filtration (**left**) and 100 kV spectrum with standard pre-filtration plus additional tin filter (Sn 100 kV) (**right**) demonstrating similar average mean intensities even with different X-ray potentials. The dotted line represents the mean energy of X-ray spectrum after passing through a 20 cm water phantom [14]. (Figure and its copyright permission courtesy of Siemens Healthineers, Erlangen, Bavaria, Germany).

In CT attenuation by the patient is high for photons with low energy ranges from 1 to roughly 30 keV such that all photons in this range will most likely interact with patient tissue [14]. Since photons of this energy range are not part of the measured signal, they are useless for imaging but contribute to patient radiation those nonetheless [14]. Therefore, photons with energy ranges not contributing to the measured signal should be eliminated or reduced in intensity wherever possible [14]. Historically, filters, e.g., aluminum, of certain thickness have been placed in the beam path permanently to absorb low energy photons to reduce patient dose level while maintaining information level of the detected signal, this is referred to as beam-hardening filtration [14]. Recently, additional pre-patient filters have been introduced to further reduce the number of low energy photons and modify the average intensities of the spectrum (spectral shaping) [14]. Tin has been evaluated as the element with the most potential for spectral shaping [14]. Therefore, in addition to existing pre-patient filtration, a movable, selectable filter made of Sn is positioned in front of the X-ray tube radiation exit window. At 100 kV this leads to a narrower X-ray tube spectrum (spectral shaping) with fewer low energy photons and higher mean energy level while maintaining similar average mean intensity compared to the standard X-ray tube voltage of 120 kV [14] (Figure 1). Ultimately, the tin filter grants for similar beam hardening effects to the standard 120 kV spectra at a significantly reduced dose.

The aim of this study was to evaluate the radiation exposure, and success of CT-guided ultra-low-dose protocol for joint injection guidance and compare these to conventional and low-dose protocols.

We hypothesize that the novel CT-guided ultra-low-dose protocol for joint injection exposes the patients to significantly less radiation than conventional and low-dose protocols, while being just as successful.

## 2. Materials and Methods

This study was conducted in accordance with the guidelines of the Declaration of Helsinki and approved by the Ethics Committee of University Hospital Erlangen under the approval number 258_18B. The Ethics Committee waived the written informed consent requirement.

We compared the radiation dose of 123 patients undergoing CT-guided joint injection featuring three different examination protocols. Furthermore, we analyzed interventional success rate with MR-arthrography images as gold standard of intraarticular or extracapsular contrast injection.

### 2.1. Patient Population

Based on their PACS archived patient protocol 123 consecutive patients who underwent CT-guided joint injection between 01/16–03/21 where retrospectively assigned to either an ultra-low-dose group (ULD), a low-dose group (LD) or a conventional group (CG). There were 60 patients in the ULD (40 men, 20 women; mean age 31 ± 13 years), 37 in the LD (22 men, 15 women; mean age 34 ± 14 years) and 26 in the CG (20 men, 6 women; mean age 37 ± 13 years). A total of 58 patients received shoulder joint injections (30 ULD, 19 LD, 9 CG) while received 65 hip injections (30 ULD, 18 LD, 17 CG).

### 2.2. CT-Guided Joint Injections

**Figure 2:**
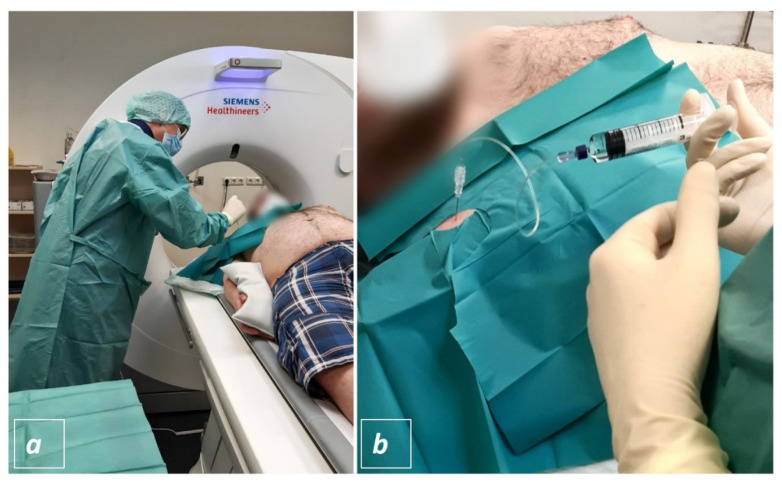
CT-guided joint injection: In shoulder injections the arm was externally rotated and this position was fixed by placing a 2 kg sandbag onto the patient’s hand. After needle pathway planning and under sterile provision the interventional radiologist advanced a spinal needle towards the joint (**a**). If the needle tip appeared to be intraarticular, 1–2 mL of Iopamidol were injected and the intraarticular distribution was confirmed by sequential CT. Thereafter, 10–20 mL of Gadopentetat-Dimeglumin were injected into the joint (**b**).

All interventions were performed utilizing a 128-slice SOMATOM go.Top CT scanner (Siemens Healthineers, Erlangen, Bavaria, Germany). In all three different protocols the patients were placed on the CT-table in supine position. In case of shoulder injection, the arm was externally rotated, and this position was fixed by placing a 2 kg sandbag onto the hand (Figure 2a).

In hip arthrography the patients were lying relaxed and with flat outstretched legs. After an initial topogram scan a sequential CT-scan of the respective joint was performed. Within this sequential dataset the joint was located, and the interventional path was planned utilizing the Guide&GO intervention tool (Siemens Healthineers, Erlangen, Bavaria, Germany). The table was moved to the corresponding position of the planed puncture site and utilizing the laser light in the gantry the estimated skin location was marked with a radio-opaque target. To verify the skin marking a single slice CT-scan was performed (Figure 2a).

If the marked skin location corresponded with the planned needle pathway, a sterile drape was placed over the target location (Figure 2a) and the skin was disinfected; if the radio-opaque target and the previously planned path did not match, target repositioning and single slice scanning were performed until satisfaction.

**Figure 3:**
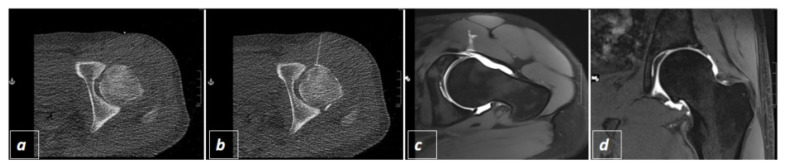
Example of CT-guided hip injection displaying the radio-opaque target as location for the needle insertion (**a**). Visualization of performed needle path and intraarticular distribution of Iopamidol following application (**b**). Visualization of intraarticular application of Gadopentetat-Dimeglumin resulting in good joint distension as demonstrated in the following MR-arthrography in transversal (**c**) and coronal (**d**) T1 weighted fat-saturated images.

**Figure 4:**
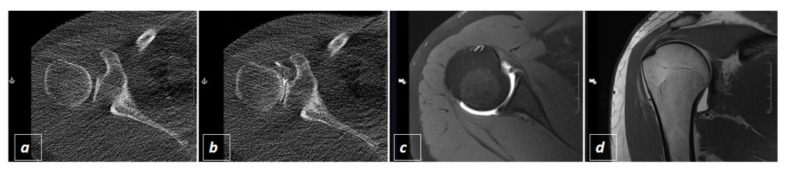
Example of CT-guided shoulder injection displaying corresponding transversal slices of the glenohumeral joint before (**a**) and after (**b**) CT-guided application of Iopamidol. Subsequent visualization of intraarticular application of Gadopentetat-Dimeglumin resulting in good joint distension as demonstrated in the following MR-arthrography in transversal (**c**) and coronal (**d**) T1 weighted images with (**c**) and without (**d**) fat-saturation.

Subsequently the interventional radiologist put on a surgical gown and stepwise advanced a 20 G spinal (Quincke Type point needle, BD^TM^, Heidelberg, Baden-Wuerttemberg, Germany) towards the joint (Figure 2a). To check the needle position, the radiologist left the CT-room and the assisting technician performed a sequential scan. If the needle tip appeared to be intraarticular the interventional radiologist injected 1–2 mL of Iopamidol (Solutrast ^®^ 250 M, Bracco Imaging Deutschland GmbH, Konstanz, Baden-Wuerttemberg, Germany) and another spiral scan was performed to check the intraarticular distribution of the CT-contrast agent (Figure 3b and Figure 4a,b). If the hyperdense contrast agent was found to be extracapsular the last two steps were repeated until the joint space was hit and contrasted. Thereafter, 10–20 mL of Gadopentetat-Dimeglumin (Magnevist ^®^, Bayer Vital GmbH, Leverkusen, North Rhine Westphalia, Germany) were injected into the joint, the needle was extracted and MR-arhtrography was performed on which joint distension and success of contrast injection could be visualized (Figure 3c,d and Figure 4c,d).

### 2.3. The Three Different Examination Protocols

**Figure 5:**
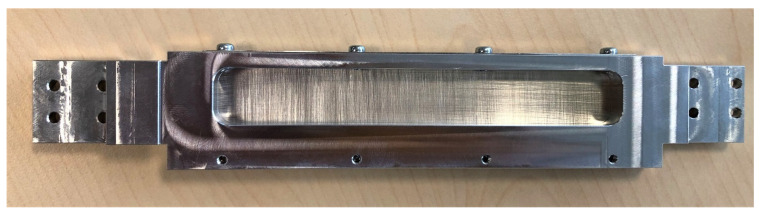
Example of a tin filter as it is incorporated in the SOMATOM go.Top scanner utilized in this research. Together with our created examination protocol featuring a tube voltage of 150 kV and a current of 5 mAs it enabled the examinations in the ultra-low-dose group. (Figure courtesy of Siemens Healthineers, Erlangen, Bavaria, Germany).

The ULD protocol consists of a topogram with a tube voltage of 100 kV and a current of 20 mAs. Each sequential scan contains 3 single slice scans with an increment and slice thickness of 3 mm, a fixed tube voltage of 150kV and a current of 5 mAs. For both, the topogram as well as the sequential scans tin filtration is applied (Figure 5). This causes spectral X-ray shaping as the tin filter removes the bulk of low energy photons that hardly contribute to image quality but increase radiation dose [15]. The tin filter can actively be added or subtracted to an examination during examination planning by the technician (within supplier given ranges of tube voltage and current). Although tin filtered images are usually reconstructed and displayed utilizing advanced modeled iterative reconstruction, this option is not given in sequential scan mode as applied in our research. All ULD CT guided injections were performed utilizing a 128-slice SOMATOM go.Top (Siemens Healthineers, Erlangen, Bavaria, Germany) scanner.

Both the LD and the CG CT-guided injections were performed utilizing a 128-slice SOMATOM Definition AS (Siemens Healthineers, Erlangen, Bavaria, Germany) scanner. Furthermore, the topograms in both examination protocols were set to a tube voltage of 120 kV and a current of 35 mAs. The X-ray tube settings in the LD examinations were set to a tube voltage of 80 kV and a current of 45 mAs, while the CG examinations were set to a tube voltage of 120 kV and a current of 50 mAs. 

### 2.4. Radiation Exposure and Interventional Success

We separately documented the dose of each complete interventional procedure including the dose of the topogram and all performed sequential scans as overall examination dose length product (DLP). Following the recommendations of the European Working Group for Guidelines on Quality Criteria for CT [16], we calculated the mean effective dose equivalent (ED) by multiplying the dose length product (DLP) with a conversion coefficient (k). In accordance with the findings published by Christner et al. [17] we applied a conversion coefficient of k = 0.015 ⁎ mSv/(mGy ⁎ cm) for the abdomen and respectively the hip interventions and k = 0.014 ⁎ mSv/(mGy ⁎ cm) for the thorax and shoulder interventions respectively.

Further, as visualized in Figure 3c,d and Figure 4c,d joint space distension and contrasting in the subsequent MRI-arthrography was evaluated for intervention success in all 123 patients.

### 2.5. Statistical Analysis

Quantitative variables are expressed as a mean ± standard deviation. The data were tested for normal distribution using the Shapiro–Wilk test. Groups were compared using Kruskal–Wallis for independent non-parametric samples. SPSS 21 (IBM Corporation, Armonk, NY, USA) was used for the analysis. A *p*-value of <0.05 was considered statistically significant. Power was calculated Sealed Envelope Ltd. 2012. Power calculator for continuous outcome superiority trial [18].

## 3. Results

There were 60 patients in the ultra-low-dose group, 30 received shoulder arthrography and 30 received hip arthrography. The mean radiation dose of the entire CT-guided joint injection (including topogram and all sequential scans) resulted in a DLP of 1.1 ± 0.39. This resulted in an effective dose of 0.0154 mSv for shoulder and of 0.0165 mSv for hip injection.

There were 37 patients in the low-dose group, 19 received shoulder arthrography and 18 received hip arthrography. The mean radiation dose of the entire CT-guided joint injection (including topogram and all sequential scans) resulted in a DLP of 5.3 ± 3.25. This resulted in an effective dose of 0.074 mSv for shoulder and of 0.08 mSv for hip injection.

There were 26 patients in the conventional group, 9 received shoulder arthrography and 17 received hip arthrography. The mean radiation dose of the entire CT-guided joint injection (including topogram and all sequential scans) resulted in a DLP of 22.93 ± 8.66. This resulted in an effective dose of 0.317 mSv for shoulder and of 0.343 mSv for hip injection.

**Figure 6:**
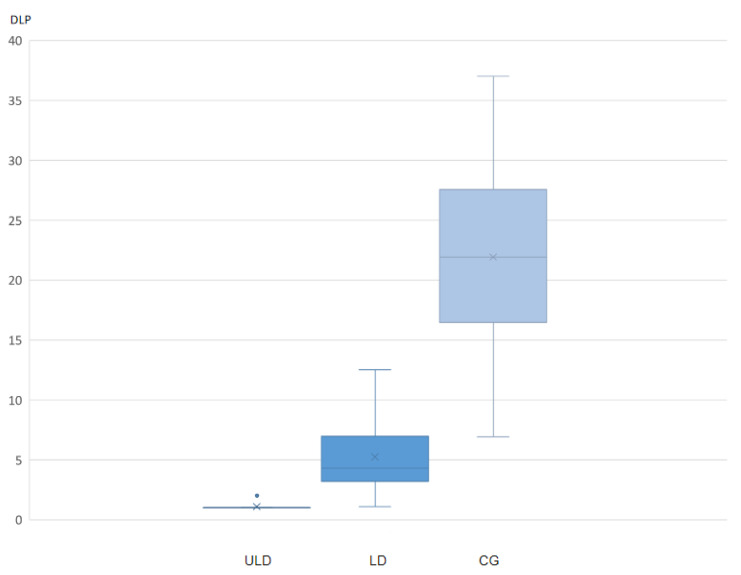
Boxplot displaying the variation between the three experimental groups concerning their radiation exposure. × displays the mean DLP values, the point displays the outlier and the whiskers display the minimum and maximum values respectively.

The Kruskal–Wallis test displayed significant differences between the DLP of the ULD versus LD (*p* < 0.01) and for the ULD versus the CG (*p* < 0.01) (Figure 6).

The ULD was successfully performed in all cases resulting in sufficient joint space distension and contrasting in the subsequent MRIs. Similarly, the CG and LD were as well performed successfully in all cases.

Power analysis demonstrated 1-β >95% for a significance level of 2.5% when performing continuous outcome superiority testing [18] for the ULD protocol against the CG as well as against the low-dose protocol.

## 4. Discussion

As demonstrated by this work, the presented novel CT-guided ultra-low-dose protocol significantly reduced patients’ effective dose in contrast to the conventional protocol and a low-dose protocol. In fact, the effective dose of a CT-guided injection utilizing the proposed protocol resulted in an average of 0.0154 millisievert for shoulder and of 0.0165 millisievert for hip injections during the entire procedure including the topogram and all interventional scans. To put this into perspective, the average annual dose of a person living in Germany corresponds to 2.1 millisiervert [19]. Therefore, the dose of our proposed ultra-low-dose CT-guided joint injection protocol equates to the natural radiation exposure of just under 3 days in Germany.

Undoubtedly, the utilization of tin filtration and aggressive X-ray spectral shaping increased the image noise, however, this did not impair the success of the joint injections as these were all performed successfully utilizing the novel presented protocol. Tin filtration has recently been utilized to drastically reduce the effective dose of various CT examinations, from virtual colonography [20] to examinations of pediatric and adolescent patients [21] or even as substitute for conventional X-ray [22]. Moreover, while Choi et al., performed CT-arthrography [12] with tin filtration and substantially reduced the radiation dose, the preparation for CT-arthrography in that study featured conventional fluoroscopy. In our investigation, we take the next logical step by trying to reduce radiation exposure from CT-guided joint injection utilizing tin filtration. Additionally, we lowered the tube current in the ULD protocol to 5 mAs which in combination with the tin filtration enabled the drastic cut in patients’ effective dose. Furthermore, imaging centers that do not have a conventional fluoroscopy unit [5] are bound to preparing MR-arthrographies utilizing CT. We expect the number of patients receiving MR-arthrographies with CT-guided joint injections (currently 9% [4]) to increase in the future and our ultra-low dose protocol has the potential to drastically reduce radiation exposure for all of these patients.

Furthermore, in contrast to the previously published low-dose protocol for CT-guided joint injection utilizing CT-fluoroscopy [7], our workflow and protocol effectively excludes any personal radiation to the interventional radiologist and mitigates wearing a lead apron. Additionally, our ultra-low-dose protocol proved to substantially outperform the CT fluoroscopy workflow as this came down to an effective dose of 0.22 mSv [7].

Although indirect MR-arthrography has been studied extensively for the shoulder, hip, knee and wrist [23,24,25,26,27] it has not been able to replace direct arthrography [28,29,30]. Although advances in musculoskeletal stress MRI [31,32] and deep learning [33] show potential in diagnosing joint pathologies, the gold standard in diagnostic workup remains direct arthrography.

One limitation of this study is the single center evaluation and the moderate amount of CT interventions in the low-dose and conventional group. To at least in parts compensate for this shortcoming, we performed power analysis which demonstrated >95% when comparing the ultra-low-dose protocol against the low-dose and the conventional protocol. Another limitation is the fact that we did not compare the radiation dose of the CT guided joint injections against conventional fluoroscopy in our work as it is not any more performed at our institution. Nevertheless, Binkert et al., demonstrated conventional fluoroscopy to result in a mean effective radiation dose of 0.0015 mSv which represents 10% of the radiation dose exposed in contrast to the by us proposed ULD protocol.

## 5. Conclusions

The presented ultra-low-dose CT-guided joint injection protocol for the preparation of MR-arthrography significantly reduces patients’ radiation dose in a way that equates to the equivalent of natural radiation exposure in Germany for just under 3 days—thereby being negligible to the patient.

## Data Availability

Data are available on request due to restrictions, e.g., privacy or ethical. The data presented in this study are available on request from the corresponding author. The data are not publicly available due to privacy.
